# Effects of Probiotics and Vitamin D_3_ Supplementation on Sports Performance Markers in Male Mixed Martial Arts Athletes: A Randomized Trial

**DOI:** 10.1186/s40798-023-00576-6

**Published:** 2023-05-16

**Authors:** Katarzyna Przewłócka, Sylwester Kujach, Piotr Sawicki, Paweł Berezka, Zofia Kinga Bytowska, Marcin Folwarski, Kondrat Kowalski, Jan Jacek Kaczor

**Affiliations:** 1 Division of Bioenergetics and Exercise Physiology, Medical University of Gdańsk, Gdańsk, Poland; 2 Department of Physiology, Gdansk University of Physical Education and Sport, Gorskiego 1, 80-336 Gdańsk, Poland; 3 Department of Human Physiology, Medical University of Gdańsk, Tuwima 15, 80-210 Gdańsk, Poland; 4 Department of Gymnastics and Dance, Faculty of Physical Education, Gdansk University of Physical Education and Sport, Gorskiego 1, 80-336 Gdańsk, Poland; 5 Department of Animal and Human Physiology, Faculty of Biology, University of Gdańsk, Wita Stwosza 59, 80-308 Gdańsk, Poland; 6 Department of Clinical Nutrition and Dietetics, Medical University of Gdańsk, 80-210 Gdańsk, Poland; 7 Masdiag Laboratory, 01-882 Warsaw, Poland; 8 Department of Animal and Human Physiology, Faculty of Biology, University of Gdańsk, Wita Stwosza 59, 80-308 Gdańsk, Poland

**Keywords:** MMA, Supramaximal sprints, Vitamin D_3_, Probiotics, Anaerobic capacity, LA utilization

## Abstract

**Background:**

Strategies targeted at the intestine microbiome seem to be beneficial for professional athletes. The gut–muscle axis is associated with the inflammatory state, glucose metabolism, mitochondrial function, and central nervous system health. All these mechanisms may affect maximal oxygen uptake, muscle strength, and training adaptation. Moreover, the positive effect of certain bacterial strains may be enhanced by vitamin D. Thus, this study aimed to assess and compare the level of selected markers of sports performance of mixed martial arts (MMA) athletes supplemented with vitamin D_3_ or probiotics combined with vitamin D_3_.

**Methods:**

A 4-week randomized double-blind placebo-controlled clinical trial was conducted with 23 MMA male athletes assigned to the vitamin D_3_ group (Vit D; *n* = 12) or probiotics + vitamin D_3_ group (PRO + VitD; *n* = 11). Repeated measures of the creatine kinase level, lactate utilization ratio, and anaerobic performance were conducted.

**Results:**

After 4 weeks of supplementation, we found lower lactate concentrations 60 min after the acute sprint interval in the PRO + VitD group when compared to the Vit D group (4.73 ± 1.62 and 5.88 ± 1.55 mmol/L; *p* < 0.05). In addition, the intervention improved the total work (232.00 ± 14.06 and 240.72 ± 13.38 J kg^−1^; *p* < 0.05), and mean power following the anaerobic exercise protocol (7.73 ± 0.47 and 8.02 ± 0.45 W kg^−1^; *p* < 0.05) only in the PRO + VitD group. Moreover, there was an improvement in the lactate utilization ratio in the PRO + VitD group compared with the Vit D group as shown by the percentage of T60/T3 ratio (73.6 ± 6.9 and 65.1 ± 9.9%, respectively; *p* < 0.05). We also observed elevated serum 25(OH)D_3_ concentrations after acute sprint interval exercise in both groups, however, there were no significant differences between the groups.

**Conclusion:**

Four weeks of combined probiotic and vitamin D_3_ supplementation enhanced lactate utilization and beneficially affected anaerobic performance in MMA athletes.

## Key Points

1. A single bout of high-intensity exercise induced an elevation in the serum 25(OH)D_3_ concentration in both groups, both before and after supplementation.
2. Combined supplementation of vitamin D_3_ with a multistrain probiotic mixture improved LA utilization after exercise.
3. MMA athletes who were supplemented with a combination of vitamin D_3_ and probiotics achieved better results, specifically in average power and total work during the first 30 s of exercise.

## Background

Mixed martial arts (MMA) is a sport that dates back to 649 BC as a part of an ancient competition. The first MMA tournaments were limited to applying as few rules as possible to resemble real fights [1]. With the development and growth in popularity, more rules have been introduced to ensure safety and equal opportunities for athletes. Moreover, certain weight classes have been introduced. Currently, a typical professional MMA fight lasts three 5-min rounds (or five rounds in the case of a title fight), separated by a 1-min break. Physical performance in MMA is characterized as interval exercise, and athletes perform high-intensity actions during the fight, interrupted by short breaks or low-intensity activity.

This sport requires energy supplied to the most extent by anaerobic metabolic pathways [2]. During high-intensity bouts of exercise, the capacities for phosphocreatine (PCr) hydrolysis and glycolysis processes are crucial to meet the maximal demand for adenosine triphosphate (ATP) resynthesis [3]. Moreover, it has been proven that the blood lactate (LA) concentration increases significantly immediately after a fight, and the values might reach up to 20.1 mmol/L, indicating high-intensity exertion with a significant proportion of anaerobic LA metabolism [4]. However, the contribution of aerobic pathways to energy yield is also elevated. It has been established that even during submaximal exercise, a certain pool of ATP is resynthesized via oxidative phosphorylation [3]. Therefore, pyruvate dehydrogenase (PDH) activity positively correlates with increased oxygen uptake, enhancing LA utilization and pyruvate oxidation [5]. The pyruvate dehydrogenase complex is composed of three subunits that require the cofactors thiamine pyrophosphate, lipoic acid, and reduced form of flavin adenine dinucleotide (FADH2), and reduced nicotinamide adenine dinucleotide (NADH) is also needed for the reaction to shift forward. This complex is a key transition point between cytosolic and mitochondrial metabolism, converting pyruvate produced by glycolysis into acetyl coenzyme-A (acetyl-CoA), which is further oxidized by the tricarboxylic acid cycle in mitochondria. Thus, training programs for MMA athletes should focus on enhancing both anaerobic and aerobic energy metabolism processes to increase the function of mitochondria, improving the proteins concentrations of substrate transport, the activity of certain enzymes of LA utilization, and glycolytic pathways.

Strategies targeted at obtaining maximal performance and skeletal muscle physiological adaptation together with the maintenance of a low risk of injuries and enhancement of regeneration processes thus represent the most important objectives among MMA athletes. In addition, an increasing number of studies indicate that the intestinal microbiome may be an indirect factor in enhancing the effect of training on physiological adaptation. This phenomenon has been called the gut–muscle axis [6, 7]. The human intestinal microbiota has been established as one of the most complex sites of the human body, with the estimated number of microorganisms exceeding 10^14^ cells. The most abundant population represents bacteria. The biodiversity and overall composition of the gut microbiota play a crucial role in maintaining homeostasis within the human body [8, 9]. It has been shown that gut microorganisms may impact the host’s nutritional status, energy metabolism pathways, and immune system function, and contribute to maintaining the integrity of epithelial cells of the gut [10]. The main mechanism through which the gut microbiome impacts muscle function is its ability to modulate oxidative stress and the inflammatory process through certain metabolic pathways, such as mammalian target of rapamycin (mTOR) kinase [11], nuclear factor kappa B (NF-kB) and Forkhead box O (FOXO) protein [12]. Moreover, some bacterial species may utilize LA, which seems to be especially important in MMA. Scheiman and coworkers have reported that animals supplemented with *Veillonella atypical* show higher LA utilization levels enhancing the Cori cycle. Improvement in the extended exercise to exhaustion time has also been observed [13]. In addition, it has been shown that serum LA crosses the epithelial barrier and is metabolized by some bacterial species to propionate, improving athletic performance [13]. It has been described that the gut–muscle axis could also be associated with glucose metabolism, mitochondrial function, and central nervous system health. All of these mechanisms may affect maximal oxygen uptake, muscle strength, and training adaptation [7]. Additionally, some studies suggest that certain strains of bacteria may decrease the blood creatine kinase (CK) level after exercise, which is a specific marker of muscle damage [14]. Moreover, in previous studies, it has been shown that certain bacterial strains such as *Bifidobacterium bifidum* W23, *Bifidobacterium lactis* W51, *Lactobacillus acidophilus* W22, *Levilactobacillus brevis* W63, and *Lactococcus lactis* W58, reduced the exercise-induced tryptophan degradation rate, limited exercise-induced drops in the tryptophan concentration and reduced incidents of upper respiratory tract infections [15]. In another study, the same probiotic strains decreased the levels of Zonulin, a marker of intestinal permeability, in feces. The authors have also observed an improvement in the exercise-induced inflammatory state in trained men [16]. These data demonstrate the benefits of some bacterial strains on sports performance via the improvement in gut homeostasis and intestinal permeability. Therefore, strategies targeted at the intestinal microbiome seem to be substantial for professional athletes.

The Food and Agriculture Organization of the United Nations (FAO) and the World Health Organization (WHO) define probiotics as “live microorganisms that, when administered in adequate amounts, confer a health benefit on the host” [17]. In light of current knowledge, it seems that the positive effect of certain bacterial strains may be enhanced by the synergistic effect of vitamin D. The discovery of vitamin D receptor (VDR) in skeletal muscle has provided evidence showing the beneficial results of cholecalciferol for proper muscle metabolism. Moreover, its role in signaling pathways appears to largely overlap with the potential intestine–muscle axis signaling pathway. The synergistic intestinal microbiota and vitamin D interaction toward muscle protein synthesis and mitochondrial function improvement may be manifested by the influence of mTOR and FOXO signaling on oxidative stress and immunological function modulation. Further research on the impact of probiotics and vitamin D_3_ on brain functions, chronic stress [18], and neuroprotection seems to be crucial, as these factors indirectly affect skeletal muscles and exercise capacities [19]. Thus, we assume that combining probiotics with vitamin D_3_ supply may have a beneficial effect on muscle function in MMA athletes. Moreover, it has been shown that reaching optimal serum concentrations of vitamin D_3_ in patients with low back pain reduced markers of oxidative stress and inflammation [20] as well as elevated the biogenesis and function of mitochondria and decreased skeletal muscle atrophy [21]. Vitamin D_3_ deficiency commonly occurs in the Polish population and is found in 85% of Poles. Currently, it is clear that an optimal serum 25-hydroxy cholecalciferol (25(OH)D_3_) concentration (≥ 30 ng/mL)is crucial for human health and sport performance. Thus, the aim of the study was to assess and compare the level of selected markers of sports performance of MMA athletes supplemented with vitamin D_3_ or probiotics combined with vitamin D_3_.

## Materials and Methods

### Study Design

**Fig. 1:**
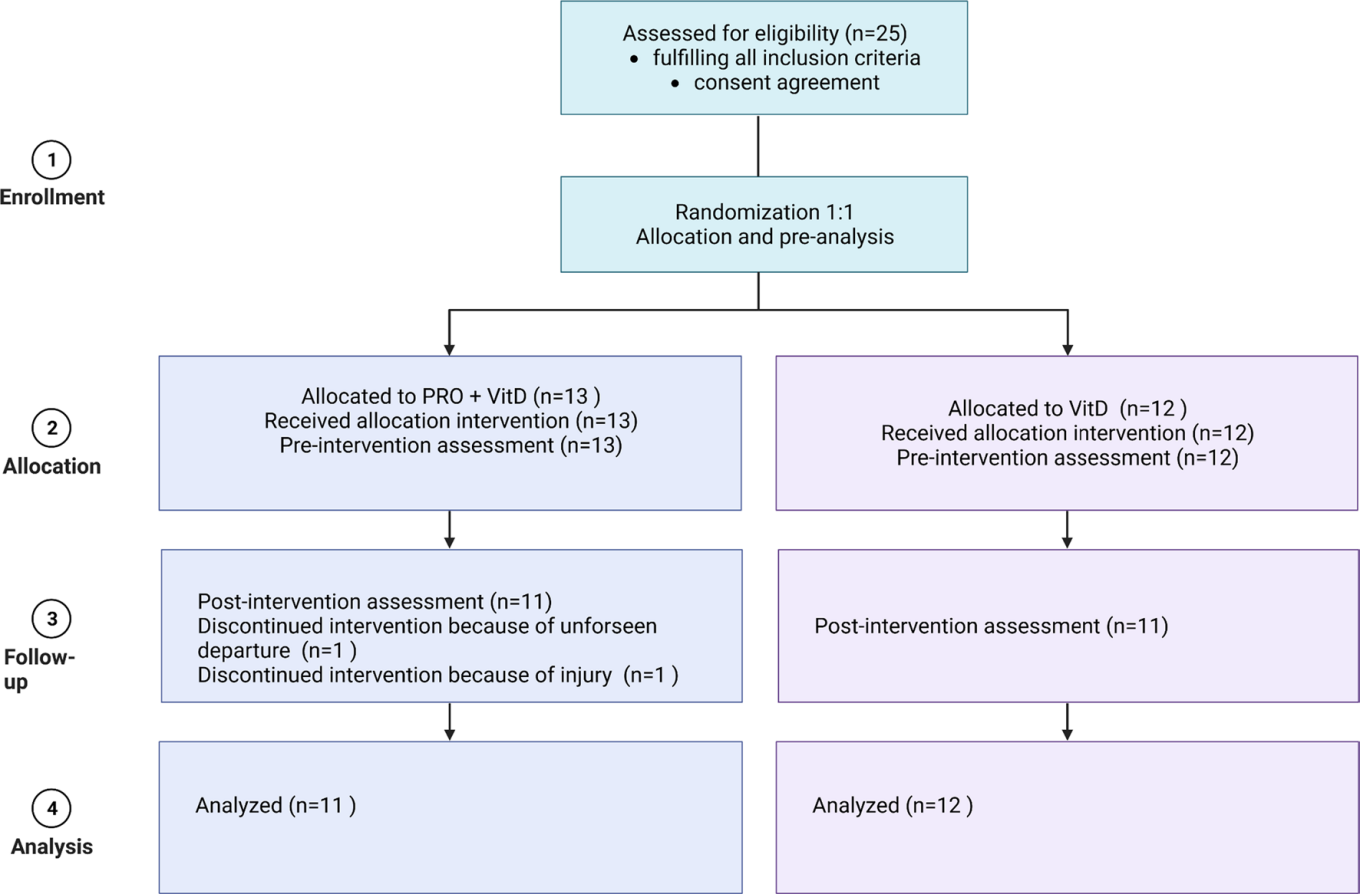
Participants flow diagram

The parallel study was designed as a randomized double-blind placebo-controlled clinical trial. Participants were randomly divided into two research conditions, with the probiotic group (PRO + VitD) receiving a multistrain probiotic together with vitamin D_3_ daily and the placebo group (Vit D) receiving a placebo preparation and vitamin D_3_ for four weeks. All athletes were Caucasian. The randomization and product allocation were conducted using an Excel random generator by an independent researcher who was not engaged in any study procedures. Athletes and investigators remained blinded until the end of the data analysis. All study procedures were performed twice: before intervention (PRE) and immediately after four weeks of intervention (POST). The protocol was approved by the Independent Bioethics Committee for Scientific Research at the Medical University of Gdańsk (No. NKNNB/643/2019-2020) in accordance with the Declaration of Helsinki. To ensure that all sufficient details were provided, Standard Protocol Items: Recommendations for Interventional Trials (SPIRIT) was adhered to [22, 23]. The study was conducted between the end of September 2020 and December 2020 in Gdansk, Poland. The research project was carried out during the autumn–winter season when sun exposure was minimal. This assumption was a priority, as we wanted to avoid any interference with the project goals by exposing MMA athletes to the sun's rays. Furthermore, according to the website Meteoblue, there were only eleven days with significant sun exposure during these three months and a couple of days in 2020. The project has been registered with Clinical Trials under the identifier NCT04759729. The study flow diagram is presented in Fig. 1.

#### Participants

Twenty-five well-trained MMA male athletes were enrolled in the study after being screened based on the inclusion and exclusion criteria. However, twenty-three of them completed the protocol. Athletes who were adults who had more than 3 years of MMA training experience, fought at least three fights and well trained a minimum of five times a week were included in the study, while participants who had a history of inflammatory bowel diseases, heart failure, antibiotic therapy within the last 2 months or a chronic injury within the previous 6 months were excluded. Some studies reported sex- and age-related differences in the gut microbiome composition [24, 25], so to reduce data variability, female sex and age < 18 years old were selected as exclusion criteria. All participants enrolled in the study were fully informed about the study’s protocol and were obligated to provide a signed informed consent form before any assessment or intervention. The sample was recruited from Gdansk and surrounding areas. All athletes who participated in the study were in the same training period throughout the clinical trial and were engaged in typical MMA workouts based on kickboxing, Brazilian jiu-jitsu, and wrestling practice, including endurance and strength sessions. Moreover, athletes were asked not to change any training or dietary habits to minimize the risk of external factors influencing obtained data.

#### Intervention

The study product was a combination of lyophilized strains of bacteria with positive effects on host health (*Bifidobacterium lactis* W51*, Levilactobacillus brevis* W63*, Lactobacillus acidophilus* W22*, Bifidobacterium bifidum* W23 and *Lactococcus lactis* W58) [15, 16], maize starch, maltodextrin, plant proteins, and hydroxypropyl ethylcellulose tablet coating. The trade name of this probiotic mixture is Sanprobi® Active & Sport (Szczecin, Poland). The total cell count was adjusted to 2.5 × 10^9^ colony forming units (CFUs) per gram (≥ 500 million CFUs in a capsule). Identical-looking capsules containing only 40 mg of maltodextrin and plant proteins were used as a placebo. The product has been tested by means of pharmacopea methods, that is culture techniques. The number of CFUs was guaranteed by the end of the shelf life, as evidenced by the use of a climatic chamber. Each pack of probiotics or placebo contained 40 capsules. At the baseline visit, all athletes received three packs of probiotics or a placebo, depending on the random allocation. Participants were also given specific instructions on how to take the product and were informed to take 4 capsules daily with meals (total daily dose of 2 × 10^9^ CFUs; 2 capsules in the morning and two capsules in the evening). The capsules were stored at room temperature in a dry place before distribution, and subjects were instructed to maintain these conditions. Athletes reported no adverse events and adhered to taking the supplements during the intervention. In addition to probiotics, athletes from both the Vit D and PRO + VitD groups received 5 ml of vitamin D_3_ supplement (in oil) containing 0.5 mg of cholecalciferol per 1 ml, which encompasses 20,000 IU, and Miglyol 812 as an excipient. Athletes were instructed to supplement 3–4 drops (3000–4000 IU) of vitamin D_3_ daily during the 4 weeks of the intervention period, together with the morning dose of probiotics.

#### Study Protocol

Participants reported twice to the laboratory: before intervention (PRE) at the baseline visit and after four weeks of the intervention period (POST) at the follow-up visit. All PRE evaluations were performed during the baseline visit, and POST evaluation were performed one day after intervention. During both visits, athletes completed an assessment of body composition, were interviewed about their dietary habits, and completed an anaerobic interval exercise—a triple Wingate test-based protocol. Furthermore, blood samples were drawn before and after the triple Wingate test to assess selected parameters of the muscle damage state and the 25(OH)D_3_ and LA concentrations. All assessments and analyses are specified in a separate section. Participants were instructed to continue their nutrition habits and the training program. Athletes were obligated to perform at least 5 specific MMA workouts per week to maintain the competitive character of the study. To assess the quality of workouts, participants were asked to note the duration of all training sessions and to evaluate the subjective feeling of fatigue in a training diaries. The study products and training diary were distributed among athletes at the baseline visit.

### Background Information

Background information was collected during the baseline visit. Athletes were asked about their health status, especially past and present injuries, diseases, medical procedures, and any mental or physical problems as well as the usage of concomitant medications.

#### Diet and Training Assessment

To assess whether any habitual changes, such as diet or training programs, might influence the gut microbiome and sports performance among athletes, a specific interview was evaluated by a qualified sports nutritionist. All athletes were obligated to perform at least 5 typical MMA trainings per week, with an average duration of 60–90 min. Typical MMA training is a mix of standing combat, grappling, ground fighting, and striking and contains strength and endurance elements. We also assessed current supplement intake with a specially prepared survey. All interviews were performed PRE and POST intervention.

#### Body Composition

Body composition was evaluated at the same time of day under the same nutritional conditions before and after the intervention. We used multifrequency bioelectrical impedance analysis (BIA) using a Tanita MC 720 analyzer to assess lean body mass (LBM), total fat mass (TFM), and body mass (BM). BIA is obtained from the measures of two parameters: resistance and reactance based on the flow of electricity through the body [21]. Before each assessment age, height, and sex were manually entered, and the participant's feet and hands were cleaned with Tanita provided tissues. During the assessment, it was necessary for the participants to stand fully erect on the measurement electrodes, hold the hand electrodes, and refrain from moving, talking, or touching the sides of the body.

### Assessments and Data Collection

#### Anaerobic Performance—Interval Exercise Session

Anaerobic performance assessments were evaluated at two-time points: the baseline and follow-up visits, in the morning, after a balanced breakfast (80 g of wheat roll, 60 g of strawberry jam, and one banana). Testing sessions were performed on a cycle ergometer (884E Sprint Bike, Monark, Sweden). Before the examination, the saddle was individually adjusted. The exercise protocol started with a 5-min warm-up at 100 watts, including two all-out sprints lasting 3–5 s in the last minute of the warm-up. Next, subjects were allowed a 3-min rest for the final preparation and then immediately started the interval exercise including three 30 s “all-out” supramaximal sprints—SIE (Wingate anaerobic test based—WAnT). The flywheel resistance equaled 7.5% of each individual’s body mass and was applied at the onset of the sprints. The interval rest periods between cycling bouts were set to 2 min. The athletes were instructed to accelerate to their maximal pedaling rate and were verbally encouraged to maintain this pedaling.

#### Venous Blood Collection and Analysis

Blood samples were collected during sports tests at two points in time, that is, PRE and POST intervention, under the same conditions. A nurse drew blood samples from the arm vein into appropriate standardized tubes containing K_2_EDTA and a coagulation activator. Each time, up to 4 ml of blood was collected. Blood samples were taken indirectly before the start of exercise tests and 30 min and 24 h after the end of the test. The blood was centrifuged at 3000 × g to separate serum and plasma. The material was placed into separately labeled microcentrifuge tubes and stored at − 80 °C for further analysis.

The collected venous blood samples were subjected to biochemical analysis. To evaluate the level of muscle damage, we determined the activity of creatine kinase (CK) in plasma through the kinetic method at 37 °C, using a RANDOX CK-NAC assay (No. CK522, Randox Laboratories LtD., Crumlin, UK) according to the “manufacturer’s instructions” with a spectrophotometer (CE9200, Cecil instruments Ltd., Cambridge, UK). The CK activity is expressed as U/l. The serum 25(OH)D_3_ concentration was assessed using the isotope dilution method by liquid chromatography coupled with tandem mass spectrometry (LC–MS/MS). All samples were prepared and analyzed using the Eksigent ExionLC analytical HPLC system with a CTC PAL autosampler (Zwinger, Switzerland) coupled with a QTRAP®4500 MS/MS system (Sciex, Framingham, MA, USA).

#### Capillary Blood Collection and Analysis

Capillary blood samples (100 µl) were taken by a qualified researcher each time sports tests were performed (PRE and POST) and collected into a sterile graduated microcapillary. The measurements were performed on fingertip capillary blood. The blood was then transferred to a microcentrifuge tube containing 500 µl 0.6 mol perchloric acid and stored at − 20 °C until the determination was carried out. Capillary blood was collected before exercise testing and at 5-time points within an hour before and after the end of the anaerobic test (TB, before; T3, 2–3 min after exercise; T15, 15 min after exercise; T30, 30 min after exercise, and T60, 60 min after the test). To determine the concentration of LA, collected capillary blood samples underwent biochemical analysis using the colorimetric kinetic method based on reaction speed. We used a lactate analyzer (Biosen C line, 173 5214 09 0045, EKF Diagnostic, Germany).

### Statistical Analysis

All raw data were analyzed using the statistical program Statistica 13.3 (StatSoft Inc., Tulsa, OK, USA). We included only full data from subjects who completed all intervention periods and all study procedures (*n* = 23). Data were previously tested for normality using the Shapiro–Wilk *W*-test. The descriptive statistics for background information and to examine the trends in the analyzed parameters with mean values and 95% confidence intervals were used. Statistical analysis was performed using two-way ANOVA test. To determine statistical significance, post hoc testing for specific differences was performed using the least significant difference (LSD) test. The statistical significance was set at *p* < 0.05.

## Results

A total of 25 MMA athletes were enrolled in the study, of whom 23 completed the protocol. Two participants in the PRO + VitD group of the study had to drop out due to unforeseen circumstances—one due to departure and the other due to injury. There were no statistically significant anthropometric differences between the groups at the baseline visit. The average age was 26.18 ± 4.05 years old in the Vit D group and 24.67 ± 6.46 years old in the PRO + VitD group. Mean body weight was 80.23 ± 9.83 kg and 81.08 ± 12 kg; fat-free mass was 71.94 ± 8.19 kg and 73.61 ± 9.33 kg; and fat mass was 8.29 ± 2.78 kg and 7.48 ± 3.85 kg in the Vit D and the PRO + VitD group, respectively. Athletes in both groups meet the criteria related to the training. The average weekly training time was 11.4 ± 3.1 h in the Vit D group and 11.8 ± 3.4 h in the PRO + VitD group. The characteristics of the MMA athletes are shown in Table 1.

**Table 1:** Participant characteristics

Participants’ information	Vit D group range	Vit D group Mean ± SD	PRO + VitD group range	PRO + VitD group Mean ± SD
Age	19–34	26.2 ± 4.0	18–40	24.7 ± 6.5
Height (cm)	168–194	179.3 ± 7.7	173–200	182.2 ± 9.3
Weight (kg)	69–98	80.2 ± 9.8	66.2–107.5	81.1 ± 12.0
FFM (kg)	59.3–87.7	71.94 ± 8.2	62.0–90.8	73.61 ± 9.3
FM (kg)	4.2–11.9	8.29 ± 2.8	2.6–16.7	7.48 ± 3.9
Years of training	5–17	10.1 ± 4.4	5–17	9.9 ± 4.0
Years of competition	3–14	7.9 ± 4.1	2–13	7.4 ± 4.1
Quantity of training (hours/week)	8.5–16	11.4 ± 3.1	9–16	11.8 ± 3.4

*FFM* fat-free mass, *FM* fat mass, *SD* standard deviation

### Effects of Supplementation on Anaerobic Performance

Anaerobic performance was assessed by applying supramaximal sprints (SIE; a triple WAnT). The obtained data indicated that post-supplementation values of total work (*W*_tot_) as well as the mean power (MP) during the first 30 s bout (WanT) were significantly different. We found an increase in *W*_tot_ (*p* < 0.05) and MP (*p* < 0.05) in the PRO + VitD group after supplementation. There were no statistically significant differences in the Vit D group (*p* > 0.05). Individuals in the Vit D group maintained their performance between pre- and post-supplementation. No positive or negative correlations were observed in this group. We found no differences between groups in the fatigue index (FI), maximal power (*P*_max_), and time to *P*_max_. There were no significant differences between the groups (Table 2).

**Table 2:** Results obtained during the first bout of supramaximal sprints

Variable	Vit D			PRO + VitD		
	PRE Mean ± SD	POST Mean ± SD	*p* value	PRE Mean ± SD	POST Mean ± SD	*p* value
*W*_tot_ [J kg^−1^]	239.34 ± 19.46	238.37 ± 14.35	0.99	232.00 ± 14.06	240.72 ± 13.38	0.04*
*P*_max_ [W kg^−1^]	10.47 ± 0.95	10.39 ± 1.22	0.96	9.80 ± 0.83	10.00 ± 0.65	0.22
*P*_max_ time [s]	6.93 ± 2.02	6.38 ± 1.56	0.53	6.67 ± 1.13	6.93 ± 1.88	0.84
MP [W kg^−1^]	7.98 ± 0.65	7.95 ± 0.48	0.99	7.73 ± 0.47	8.02 ± 0.45	0.04*
FI [w/kg/s]	0.20 ± 0.04	0.18 ± 0.06	0.06	0.17 ± 0.05	0.17 ± 0.04	0.97

The bold text indicates a significant difference (*p < 0.05) between PRE and POST supplementation in the PRO + VitD group

*PRE* before supplementation, *POST* after supplementation, *W*_tot_ total work, *P*_max_ maximal power, *P*_max_* time* time to obtain max power, *MP* mean power, *FI* fatigue index (decrease rate of *P*_max_), *SIE* supramaximal sprints

### Effects of Supplementation on LA

**Fig. 2:**
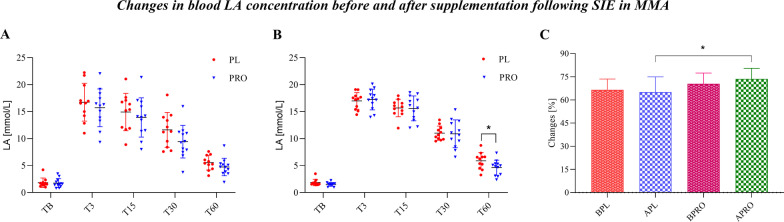
**A** The concentration of LA before and in 4-time points within an hour after the end of SIE in both groups before supplementation (PRE). **B** LA concentration after 4 weeks of supplementation (POST) was lower in the Vit D vs. PRO + VitD group 60 min after SIE (*p < 0.05; LSD). **C** The ratio of LA utilization (T60/T3) expressed as a percentage (*p < 0.05; LSD)

The blood LA concentration in both groups before supplementation, and exercise as well as after SIE was not significantly different between the groups. The LA concentration before exercise (TB) was 1.82 ± 0.87 mmol/L (Vit D) and 1.77 ± 0.82 mmol/L (PRO + VitD) and increased at 3 min (T3; Vit D 16.74 ± 3.50 mmol/L and PRO + VitD 15.71 ± 3.49 mmol/L after SIE and then decreased to values of 5.54 ± 1.36 mmol/L and 4.73 ± 1.63 mmol/L within 60 min (T60) in the Vit D and PRO + VitD groups, respectively (Fig. 2A). After four weeks of supplementation we found a significantly higher concentration of LA in the Vit D group (5.88 ± 1.55 mmol/L) than in the PRO + VitD group (4.73 ± 1.63 mmol/L) 60 min (T60) after SIE (*p < 0.05; Fig. 2B). In addition, we also observed that LA was more effectively utilized in the PRO + VitD group (73.6 ± 6.9%) compared with the Vit D group (65.1 ± 9.9%) from the highest concentration at T3 to T60 after SIE. The rate of LA oxidation is shown as the percentage of the T60/T3 ratio (*p < 0.05; Fig. 2C).

### Effects of Supplementation on the Serum 25(OH)D_3_ Concentration

**Fig. 3:**
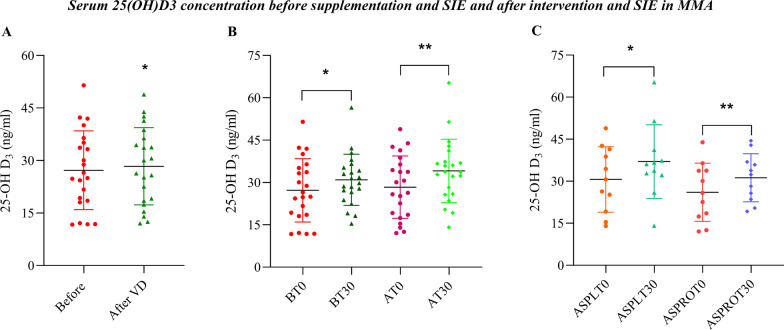
**A** The concentration of 25(OH)D_3_ before (PRE) and after 4 weeks (POST) of vitamin D_3_ supplementation (*p < 0.05; LSD). **B** Serum 25(OH)D_3_ concentration in MMA at BT0 (PRE; before supplementation and exercise) compared to BT30 (before supplementation after 30 min of SIE) and AT0 (POST; after intervention before exercise) versus AT30 (after supplementation 30 min after SIE). Data are expressed as ng/mL (*p < 0.05; LSD). **C** The concentration of 25(OH)D_3_ after 4 weeks of combined vitamin D_3_ and probiotics supplementation (*p < 0.05; LSD) before and 30 min after SIE; ASPLT0-after supplementation of vitamin D_3_ before SIE; ASPLT30-after supplementation of vitamin D_3_ after 30 min SIE; ASPROT0-after combined supplementation vitamin D_3_ with probiotics before SIE, ASPROT30-after combined supplementation vitamin D_3_ with probiotics after 30 min SIE

The mean value of all MMA athletes regarding the serum 25(OH)D_3_ concentration before the supplementation period was 27.22 ± 11.23 ng/mL, and after four weeks of supplementation with an average amount of 3500 IU/day vitamin D_3_, the serum 25(OH)D_3_ concentration was 28.35 ± 11.03 ng/mL (*p < 0.05; Fig. 3A). To our surprise, despite vitamin D_3_ supplementation at a dose of 3 500 IU/day, the serum concentration of 25(OH)D_3_ was still below the minimum (30 ng/mL). On the other hand, in both pre-supplementation groups, the serum concentration of 25(OH)D_3_ was significantly elevated 30 min after SIE (*p < 0.05 BT0 vs. BT30; Fig. 3B). Moreover, after 4 weeks of intervention and 30 min after the SIE, there was an even higher increase in 25(OH)D_3_ in serum (**p < 0.001 AT0 vs. AT30; Fig. 3B).

Furthermore, the release of 25(OH)D_3_ into the bloodstream, 30 min following SIE after 4 weeks of supplementation was higher, in the Vit D group (*p < 0.001; ASPLT0 vs. ASPLT30; 6.4 ng/mL) as well as in the PRO + VitD group (**p < 0.01; ASPROT0 vs. ASPROT30; 5.1 ng/mL). We noticed a trend toward a higher release of 25(OH)D_3_ after 30 min of SIE following supplementation in the Vit D group than in the PRO + VitD group; however, the differences did not reach statistical significance (Fig. 3C).

### Effects of Supplementation on CK Activity

We observed that plasma CK levels were slightly higher 24 h after SIE before the supplementation period (PRE) as well as after supplementation (POST) compared with levels before exercising in both groups. However, we noticed no statistically significant differences between the Vit D and the PRO + VitD groups after the supplementation period. In addition, the changes in CK activity after supplementation were not significant before SIE in the Vit D and PRO + VitD groups or 24 h after SIE in either group. The plasma CK activities are presented in Table 3.

**Table 3:** Effects of supplementation on plasma creatine kinase level

Variable	Vit D			PRO + VitD		
	PRE Mean ± SD	POST Mean ± SD	*p* value	-PRE Mean ± SD	POST Mean ± SD	*p* value
Before SIE	154.6 ± 103.7	195.0 ± 183.4	0.969	152.2 ± 143.1	269.4 ± 214.7	0.726
24 h After	316.0 ± 142.5	317.3 ± 255.9	0.999	447.8 ± 347.9	339.2 ± 270.6	0.911

*PRE* before supplementation*, POST* after supplementation*, SIE* supramaximal sprints*, SD* standard deviation Statistical significant (*p* < 0.05)

## Discussion

To the author’s knowledge, this is the first study to investigate a multistrain probiotic mixture combined with vitamin D_3_ in the mixed martial arts (MMA) athlete population. The current study examined the influence of 4 weeks of probiotic and vitamin D_3_ supplementation on anaerobic performance, LA utilization, and muscle damage. Athletes tolerated both supplements well and did not experience any side effects. We found that a single bout of high-intensity exercise elevated the serum 25(OH)D_3_ concentration in both groups, both before and after supplementation, which is in agreement with recently published data [26, 27]. Moreover, combined vitamin D_3_ with multistrain probiotic mixture supplementation also showed improvement in LA utilization after SIE (between 15 and 30 min) compared to the VIT D group. This may support the assumption that changes in the gut microbiome caused the enrichment of species able to metabolize LA and thus enhanced its faster utilization [28]. MMA athletes supplemented with vitamin D_3_ and probiotics achieved better results in anaerobic performance tests. We found a beneficial effect on average power and total work during the first 30 s of SIE.

Recent studies have established the crucial role of the gut microbiota composition in immune function [29–31] and brain health [32, 33], which may be indirect factors influencing physiological adaptation to training. However, the potential beneficial connection between intestinal microbiota composition, muscle function, and sports performance is not clearly understood. Increasing evidence has confirmed the importance of the interplay between gut homeostasis, inflammatory processes, and skeletal muscle adaptation to training, which we demonstrated in our previous review [7].

Short-term, high-intensity interval exercises are essential to an MMA athlete’s training program. It is well known that the metabolic response to this type of physical effort leads to the accumulation of LA and hydrogen in skeletal muscles and blood circulation, which as a consequence, may impair physical performance [34]. It was suggested that intramuscular LA accumulation and the associated pH decrease among muscle cells due to the development of fatigue during exercise exert a detrimental effect on glycolytic energy provision and potassium release [35]. Therefore, athletes’ ability to remove or metabolize LA seems to be a crucial factor influencing sports performance. Moreover, the blood LA response to exercise can be a useful factor in assessing exercise capacity. In our study, we observed that 4 weeks of probiotic supplementation contributed to a higher rate of LA utilization after SIE than at baseline. In contrast, no changes in the Vit D group occurred. In the PRO + VitD group, the blood LA concentration decreased significantly within 1 h after SIE to the value obtained 3 min after exercise. The utilization rate at baseline was 66.5% in the Vit D group and 70% in the PRO + VitD group. After the supplementation period, the utilization rate was approximately 66.5% in the Vit D group and 74% in the PRO + VitD group. Significant changes were observed 15 min after the SIE. The LA produced during high-intensity exercise mostly comes from fast-twitch muscle fiber, which consume large amounts of glucose to generate energy, and it is mainly removed by slow twitch muscle fiber. According to Brooks’ theory, improvement in LA utilization may be caused by the enhancement of clearance complex processes engaging lactate-specific enzymes and transporters. In addition, LA may be used as an additive energy source as well as a gluconeogenesis substrate that enhances glycolytic processes [36].

In light of current knowledge, the composition of the intestinal microbiome of athletes differs from that of inactive people, mainly due to the greater abundance of bacterial species, biodiversity, and a higher proportion of some bacterial species such as *Veillonella*, *Bacteroides*, *Akkermansia*, *Methano brevi bacteria* and *Prevotella* [37, 38]. Sheiman et al. indicated, that the relative abundance of *Veillonella* strains increases in long-distance runners after a marathon. In addition, another group showed that each gene in the major metabolic pathway that metabolizes LA to propionate was more abundant after exercise than before exercise and that LA can overcome the epithelial barrier to the intestinal lumen [28]. The same researchers isolated *Veillonella atypica* from marathon runners’ stool samples and transplanted them into mouse intestines. As a result, there was a significant increase in running time on a treadmill to exhaustion [28]. According to these data, it is very likely that exercise-induced LA is released from muscle cells into the bloodstream and then crosses the epithelial barrier, where it is metabolized to propionate by *Veillonella atypical* and other bacterial species using it as the sole carbon source. LA is converted to propionate via the methylmalonyl-CoA pathway [39], and lactate dehydrogenase (LDH), a crucial enzyme engaged in LA metabolism, is present in a phylogenetically diverse group of bacteria [28]. Taken together, some bacteria may improve physical performance via a microbial-encoded enzymatic process, which enhances LA utilization and delivers extra energy and gluconeogenesis substrates.

In our study, we observed improved LA utilization after 4 weeks of multistrain probiotic mixture supplementation, including some species that metabolized it. Therefore, it is very likely that changes in the gut microbiome caused the enrichment of species able to metabolize LA and thus enhanced its utilization. A similar effect was observed in the study by Huang and coworkers, where 6 weeks of *Lactiplantibacillus plantarum* TWK 10 supplementation significantly improved LA accumulation both during exercise and after 90 min in the recovery period in healthy adults. Researchers found that the beneficial effect of Lactiplantibacillus*plantarum* TWK 10 supplementation on LA utilization was stronger during the recovery phase [40]. Interestingly, a previous study indicated that mice supplemented with the same bacterial strain showed positive changes in the gut microbiome composition, significantly affecting the relative abundances of *Bacteroidetes* and *Firmicutes* [40]. Moreover, *Lactiplantibacillus plantarum* TWK 10 intake resulted in a decrease in LA blood accumulation after a 15-min swimming trial [41]. Another animal model study showed that a multistrain probiotic mixture significantly decreased the postexercise LA concentration in horses subjected to athletic activity. These findings also suggest that probiotics may promote physical performance by enhancing short-chain fatty acid (SCFA) production, which is used by muscles as an energy source instead of carbohydrates [42]. Scientific data confirm that the *Lactobacillus* genus may alter the hindgut pH and induce the proliferation of LA-utilizing bacteria such as *Veillonell asp.* Thus, the source of energy usage is modified during exercise [42]. Our data are in line with Lighi and coworkers. However, in contrast to a study in triathlon athletes, 8 weeks of supplementation with *Lactiplantibacillus plantarum* PS 128 did not affect the LA concentration, although it did beneficially affect physical performance and inflammatory markers after exercise [43].

According to current knowledge, it seems that intestinal microbiota-targeted strategies may improve training parameters as well as increase training capabilities. In our study, 4 weeks of multistrain probiotic supplementation improved anaerobic performance. We found an improvement in average power and total work during the first bout of SIE in the PRO + VitD group, while no significant changes were found in the Vit D group. Similar results were shown by the study of Jager et al., who supplemented men exercising recreationally with *Weizmannia coagulants* GBI-30, 6086 (BC30). Probiotic ingestion prevented the decline in peak power in the probiotic group, but no changes occurred in strength or vertical jump. The authors showed that probiotic supplementation significantly decreased postexercise CK levels [14]. Interestingly, no muscle damage as measured by CK was observed in our study. Although we observed an increase in the plasma CK concentration 24 h after SIE, it was not high enough to indicate muscle damage. Moreover, we did not detect any differences in plasma CK levels between the groups. The lack of significant changes in CK levels may be due to the large standard deviations in both groups. It is well known that CK levels are elevated after excessive training and may reach thousands of units per liter. However, substantial individual variability was reported. The magnitude of muscle damage marker differences may be influenced by the intensity and duration of exercise, training state of athletes, sex, genetic predisposition, distribution of muscle fiber type, kind of muscle contraction, and type of exercise [44]. A single bout of the 30-s Wingate test triggered muscle damage manifested by elevated CK concentration in non-athlete individuals [45]. In our study, SIE did not increase muscle damage measured by CK levels in MMA athletes. It is possible that this kind of exercise, which resulted in high LA concentrations, as well as high self-reported fatigue, was not sufficient to damage the skeletal muscle tissue among combat sports athletes. Perhaps this may be due to the high physiological adaptation to this exercise type and intensity. It is worth noting that scientific reports investigating the effect of probiotics on CK concentration are ambiguous. Some animal and human model studies have supported the hypothesis that some bacterial strains may enhance muscle regeneration by attenuating CK levels and thus muscle damage [14, 41, 43]. Our results are in line with the study by Jager et al., where *Streptococcus thermophilus* FP4 and *Bifidobacterium breve* BR03 supplementation improved the maximal voluntary isometric peak torque and flexed arm angle after a damaging workout without plasma CK induction among resistance-trained male participants [46]. Similarly, supplementation of Weizmannia coagulants GBI-30, 6086 (BC30) in soldiers positively affected muscle strength and attenuated the inflammatory response during intense training (mean jumping power) but did not influence the level of CK [47].

Concerning anaerobic performance, a similar effect was reported by Huang et al. in triathletes after 8 weeks *Lactiplantibacillus plantarum* PS128 supplementation. According to our results, the probiotic intervention improved the mean peak power, mean power, and fatigue index. Moreover, the authors showed a positive effect on oxidative stress markers, CK, and proinflammatory cytokine plasma concentrations [43]. Interestingly, the test was performed on the same day as the official triathlon competition, after 6 h of rest. It is well known that excessive physical exercise due to neuromuscular deficits is responsible for forcing transmission impairment. In this scenario, a lower than average positive force and a slower rate of force development were observed [48]. Thus, some bacterial strains may be a potential alternative ergogenic aid, contributing to the maintenance of mean power and the fatigue index (FI) via the positive effect on blood metabolites and thus on muscle physiological adaptation. There is a high probability that this effect may be enhanced through proper vitamin D_3_ concentration (> 30 ng/mL), which we found in both PRE and POST supplementation after SIE. Therefore, we assume that regular physical exercise may have a beneficial impact on nervous system and muscle health. Thus, the synergistic effect of probiotics and vitamin D_3_ could have particular importance in this area.

Recently, data from our laboratory reported that a serum vitamin D_3_ deficit is associated with oxidative stress, negatively affecting mitochondrial function and skeletal muscle metabolism [19]. Moreover, the discovery of vitamin D receptor (VDR) in skeletal muscle provided evidence showing the beneficial effects of cholecalciferol on proper muscle metabolism, and its role in signaling pathways appears to largely overlap with the potential intestine–muscle axis signaling pathway. Thus, we aimed to detect whether cosupplementation of vitamin D_3_ and probiotics is favorable to sports performance and if this strategy is more effective than supplementation of vitamin D_3_ alone. In the current study, we found a significant increase in the serum 25(OH)D_3_ concentration 30 min after the entire SIE in both groups compared to the initial value. Our data are consistent with a study by Dzik et al., showing a significant increase in serum 25(OH)D_3_ concentrations after an acute 30 s WAnT in young, trained boys. Moreover, this elevation positively correlated with fat-free mass (FFM), suggesting the release of 25(OH)D_3_ from the muscle tissue [26]. Interestingly, a recent study indicated that a 20-week resistance training program increased the plasma 25(OH)D_3_ concentration from 42.4 to 51.2 nmol/L and induced *CYP27B1* gene expression, having a positive impact on VDR regulation. In this investigation, no vitamin D_3_ supplementation was implemented [26]. Thus, it is highly probable that skeletal muscles may store and release 25(OH)D_3_. Our data suggest that physical exercise may trigger the release of some metabolites of vitamin D_3_ into the blood circulation. It seems that the training process may enhance this increase. In our study, we observed a slight increase in 25(OH)D_3_ in blood serum after 4 weeks of supplementation. MMA athletes reached the minimum recommended value, but their levels were still not optimum. We suggest that the doses of vitamin D_3_ were too low to induce physiological effects. Thus, no spectacular changes in MMA athletes were observed. It was also shown that seven months of vitamin D_3_ supplementation (1200 IU daily) had no effect on calcium, parathormone, cortisol, and testosterone blood concentrations, as well as hand grip strength among Estonian soldiers [49].

Similarly, no effect on peak power output was reported by Hew-Bulter et al., who performed a 12-week supplementation of 4000 IU vitamin D_3_ in basketball players [50]. To summarize, vitamin D_3_ recommended doses for healthy, active adults are probably too low for a population of elite athletes. Moreover, because of the potential high muscle usage of vitamin D_3_, athletes seem to be at risk of vitamin D_3_ deficiency. It appears that supplementation of vitamin D_3_ combined with probiotics may potentially result in enhanced serum vitamin D_3_ concentrations. The potential mechanism is associated with the similarity of lipid and vitamin D_3_ absorption in the gut. It seems that some microorganisms, via improved intestinal emulsification of lipids can enhance vitamin D_3_ gut absorption. This hypothesis was confirmed by Castagliuolo et al., who supplemented mice with *Lacticaseibacillus paracasei* DG and oil-based cholecalciferol. The authors observed that supplementation resulted in maintaining adequate vitamin D_3_ levels in the blood [51]. This preclinical study suggests that the coadministration of probiotics and vitamin D_3_ may be more effective in preventing and treating vitamin D_3_ deficits.

### Limitations of the Study

A possible study limitation was the lack of diet standardization; even though participants were asked not to change their previous eating habits (to avoid the influence of diet on the gut microbiome), participants also declared that they were not taking any medications, not drinking alcohol, and not smoking during the study and 1 month before the study. There was no true control group in this study either. It is also possible that the proposed exercise protocol was insufficient to damage the skeletal muscle tissue in combat sports athletes. However, the SIE modality resulted in high LA concentrations and a high self-reported fatigue score reflecting sport fighting conditions. Finally, on average, 3500 IU/day of vitamin D_3_ supplementation seemed to be too low for professional MMA athletes.

## Conclusion

We found that exercise increased serum concentrations of 25(OH)D_3_ in both groups before and after supplementation. In addition, the combined intake of vitamin D_3_ and a probiotic mixture containing several strains showed an improvement in LA utilization after SIE in the supplemented group. This finding may support the notion that changes in the gut microbiome led to an enrichment of species that can metabolize LA, allowing for more rapid utilization of lactate. MMA athletes supplemented with combined vitamin D_3_ and probiotics showed total work and mean power improvement in the anaerobic test. However, further studies should be conducted with higher vitamin D_3_ doses, gut microbiome composition analysis, serum vitamin D binding protein determination, and different exercise types and intensities to better understand the mechanisms involved in the physiological adaptation.

## Data Availability

The datasets generated during and/or analyzed during the current study are available from the corresponding author on reasonable request.

## Abbreviations

MMA: Mixed martial arts
PCr: Phosphocreatine
LA: Lactate
PDH: Pyruvate dehydrogenase
FADH2: Reduced form of Flavin Adenine Dinucleotide
NADH: Reduced form of Nicotinamide Adenine Dinucleotide
mTOR: Mammalian target of rapamycin
NF-kB: Nuclear factor kappa B
FOXO: Forkhead box O protein
FAO: The Food and Agriculture Organization of the United Nations
WHO: The World Health Organization
VDR: Vitamin D receptor
25(OH)D_3_: 25-Hydroksy cholecalciferol
PRO + VitD: Probiotic plus vitamin D_3_ group
Vit D: Vitamin D_3_ group
PRE: Before intervention
POST: 4 Weeks after intervention
SPIRIT: Standard Protocol Items: Recommendations for Interventional Trials
CFUs: Colony forming units
LBM: Lean body mass
TFM: Total fat mass
BM: Body mass
WAnT: Wingate anaerobic test
SIE: Supramaximal sprints based on WAnT
TB: Time point before exercise
T3: 2–3 Minutes after exercise
T15: 15 Minutes after exercise
T30: 30 Minutes after exercise
T60: 60 Minutes after exercise
FFM: Fat-free mass
*W*_tot_: Total work
MP: Mean power
*P*_max_: Maximal power
*P*_max_ time: Time to obtain maximal power
FI: Fatigue index
BT0: Before supplementation, before exercise
BT30: Before supplementation, 30 min after exercise
AT0: After supplementation, before exercise
AT30: After supplementation, 30 min after exercise
CK: Creatine kinase
LDH: Lactate dehydrogenase
SCFA: Short-chain fatty acid
